# Biochemical and biophysical characterization of pyruvate kinase M2 activation by L-serine

**DOI:** 10.1186/2049-3002-2-S1-P12

**Published:** 2014-05-28

**Authors:** Barbara Chaneton, Finn Holding, Adam Hold, Jiska Van Der Reest, Gabriele Marciano, Marc O’Reilly, Eyal Gottlieb

**Affiliations:** 1 Cancer Research UK, The Beatson Institute for Cancer Research, Glasgow, UK; 2 Astex Pharmaceuticals, Cambridge, UK

## 

We have shown that serine binds to an amino acid binding pocket located in the A domain of each PKM2 monomer, but the molecular mechanism by which it modifies PKM2 activity is still unknown, especially regarding the switch from dimer to tetramer. While fructose 1,6 bisphosphate (FBP) activates PKM2 by promoting tetramerization, it is not clear if serine is able to modulate the oligomerization status of PKM2 since it binds to a completely different region of the protein. In order to understand whether serine can induce PKM2 tetramerization similarly to FBP, we looked at the oligomeric state of recombinant purified PKM2 under native conditions by HPLC-UV SEC (size exclusion chromatography). We tested the effects of increasing concentrations of L-Serine on the oligomeric state of PKM2. Additionally, in order to understand whether PKM2 activation by serine may be mediated or affected by the oligomeric state of the protein, we tested if serine is able to activate PKM2 even when tetramerization is impaired. For that purpose, we generated several monomer-monomer and dimer-dimer interface mutants aimed to disrupt the ability of PKM2 to tetramerize. By using HPLC-UV SEC we have been able to see changes in the oligomeric state of wild type PKM2 and we have confirmed that FBP induces its tetramerization. We have generated the S437Y PKM2 mutant that cannot bind FBP and an additional, H464A mutant that cannot bind serine. Our *in vitro* activity assays indicate that PKM2 binding to FBP is required also to fully activate the protein in the presence of serine. We also determined the oligomeric state of the S437Y and H464A mutants in the presence of different concentrations of serine and FBP to investigate the possibility of a dependency between the two mechanisms of activation and how it relates to the oligomeric state of PKM2.

